# Point Cloud Wall Projection for Realistic Road Data Augmentation

**DOI:** 10.3390/s24248144

**Published:** 2024-12-20

**Authors:** Kana Kim, Sangjun Lee, Vijay Kakani, Xingyou Li, Hakil Kim

**Affiliations:** 1 Department of Electrical and Computer Engineering, Inha University, Incheon 22212, Republic of Korea; 2 EV Charger Development Team, Hyundai KEFICO Corp., Gunpo 15849, Republic of Korea; 3 Department of Integrated System Engineering, Inha Universiy, Incheon 22212, Republic of Korea

**Keywords:** LiDAR, point cloud, synthetic data, data augmentation, object detection

## Abstract

Several approaches have been developed to generate synthetic object points using real LiDAR point cloud data for advanced driver-assistance system (ADAS) applications. The synthetic object points generated from a scene (both the near and distant objects) are essential for several ADAS tasks. However, generating points from distant objects using sparse LiDAR data with precision is still a challenging task. Although there are a few state-of-the-art techniques to generate points from synthetic objects using LiDAR point clouds, limitations such as the need for intense compute power still persist in most cases. This paper suggests a new framework to address these limitations in the existing literature. The proposed framework contains three major modules, namely position determination, object generation, and synthetic annotation. The proposed framework uses a spherical point-tracing method that augments 3D LiDAR distant objects using point cloud object projection with point-wall generation. Also, the pose determination module facilitates scenarios such as platooning carried out by the synthetic object points. Furthermore, the proposed framework improves the ability to describe distant points from synthetic object points using multiple LiDAR systems. The performance of the proposed framework is evaluated on various 3D detection models such as PointPillars, PV-RCNN, and Voxel R-CNN for the KITTI dataset. The results indicate an increase in mAP (mean average precision) by 1.97%, 1.3%, and 0.46% from the original dataset values of 82.23%, 86.72%, and 87.05%, respectively.

## 1. Introduction

The accurate perception of a vehicle’s surrounding environment is essential for advancing autonomous driving technologies, as it enables the vehicle to safely navigate complex and dynamic road scenarios. Among various visual sensors, 3D LiDAR is particularly effective for capturing detailed spatial information, providing valuable insights into the position and distance of surrounding objects. However, acquiring large-scale, high-quality LiDAR data remains challenging due to the high cost of LiDAR sensors and the labor-intensive nature of 3D data labeling.

To address the demand for robust training data, recent synthetic data generation approaches have been explored, but they face key limitations: synthetic LiDAR point cloud generation, in most cases, demands intense computing power, and rendering-based methods struggle to represent sparse point clouds of distant objects accurately. Such limitations reduce the utility of synthetic data in training deep learning models for autonomous driving, particularly for scenarios involving complex interactions or specific orientations, such as vehicle platooning.

This study proposes a novel approach to generate synthetic object points from real point cloud data rather than synthetic data, improving the realism of distant object representations. Specifically, a “point-wall” technique is introduced to compensate for the excessive loss of details in distant objects, enhancing their shape fidelity. With the use of spherical point projection, the generated distant points are intended to resemble real LiDAR point clouds since the synthetic points are modified from real LiDAR point clouds as opposed to other simulators. The notable difference between the real-world point clouds and the generated ones can be attributed to several aspects, including the return losses, which are not considered in the current study. Additionally, a pose determination module is integrated to capture realistic object orientations, enabling the representation of platooning vehicles on the road—an aspect previously unaddressed in virtual object generation.

The key contributions of this work are as follows:

- To enhance the representation of distant objects using the refinement of spherical point projection without the need for complex extrapolation techniques.
- To prevent excessive loss in virtual objects’ details, ensuring the shapes of distant objects resemble real sensor data more closely via the point-wall method.
- To accurately depict the orientation of synthetic object points, supporting realistic platooning scenarios on roadways using the pose determination module.

The remainder of this paper is organized as follows. Section 2 outlines the research literature relevant to 3D object detection and data generation aspects. Section 3 describes the proposed methodology and its attributes, such as position estimation, object generation, and synthetic annotation. Section 4 provides information about the experimental environment, the dataset, synthetic LiDAR point cloud generation, deep learning model training, and relevant quantitative and qualitative results. Section 5 states the shortcomings of the proposed method and proposes potential future research based on the presented methodology, and Section 6 concludes the research study.

## 2. Related Works

Several methods are being developed in the context of LiDAR-based vehicle perception to improve detection performance, mainly by restoring lossy point cloud data to synthetic points. The main aim of these methods is to create synthetic points to enhance data, owning to a loss of 3D point cloud data and a lack of objects. However, because these methods require considerable computation, they are difficult to operate in an embedded environment. Data generation using well-designed simulators is an another way to overcome the shortage of point cloud data. A few methods, such as that proposed by Esmoris et al. [1], tackle this by exploring virtual laser scanning (VLS), demonstrating that simulated data can achieve comparable results to real-world data. VLS represents a scalable and cost-effective alternative, although further integration with dynamic scenarios is needed for its real-world application. VLS methods are often referred to as approaches to generating synthetic point clouds, including the method used in this study. Also, Beltran et al. [2] obtained dense point cloud data using LiDAR sensors and generated point cloud data for the desired rendering environments, such as simulators. There is a large focus on developing a method for collecting and using LiDAR data from 3D games [3] or a method for rendering and using data from the LiDAR sensor of an autonomous driving simulator platform [4,5]. The simulator’s LiDAR sensor data are ideal as they lack real noise, so the rendered results exhibit a large difference compared with the real data. Also, synthetic LiDAR point cloud generation techniques using the GAN [6] model can be used as augmentation techniques for datasets. In addition, Yin et al. [7] enhanced the DART model with Monte Carlo-based methods, enabling precise satellite LiDAR simulations and facilitating data fusion with other remote sensing modalities. Furthermore, Yin at al. [8] extended LiDAR waveform simulation to multi-pulse systems and introduced new methods for simulating photon-counting data and solar noise in diverse configurations. Gastellu et al. [9] introduced a comprehensive 3D radiative transfer model that simulates the interaction between Earth, the atmosphere, and sensor specifications for remote sensing applications. Also, Gastellu et al. [10] expanded on DART by integrating LiDAR waveform simulation, demonstrating its versatility across various landscape and atmospheric configurations and its ability to model multi-scattering effects. Similarly, Yang et al. [11] presented DART-Lux, a novel LiDAR modeling approach that enhances simulation efficiency using a bidirectional path-tracing algorithm, while comparing different tracing methods for improved accuracy. Further Yang et al. [12] validated the DART-Lux model using real GEDI and ICESat2 data, quantifying inconsistencies in height measurements and incorporating atmospheric effects for more accurate LiDAR simulations in large-area landscapes.

LiDAR-Aug [13] was developed to generate synthetic object point clouds by generating point cloud data from synthetic objects. It expresses the point distribution of the real object’s data but is vulnerable when generating distance points for the real object data in their version of synthetic point clouds. To address this, Xiao et al. [14] proposed SynLiDAR, a large-scale synthetic dataset with annotated point clouds, and the Point Cloud Translation (PCT) method to bridge the gap between synthetic and real point clouds. While effective in improving transfer learning strategies on 32 custom-built semantic classes, it still faces challenges in generating data for rare object categories because of data imbalances (little to no samples in the rare classes). Xiang et al. [15] built on this by introducing a data augmentation method using generative models like L-GAN to enhance rare classes in LiDAR point cloud datasets. This method effectively balances class distribution, improving recognition performance across both minority and majority classes. However, while generative models address data imbalance, they still rely on high-quality real data for model training.

In contrast, D-Aug [16] retrieved objects and integrated them into dynamic scenarios, taking into account the continuity of these objects across successive frames. However, D-Aug suffers from post-insertion occlusion due to complicated and cluttered situations arising after the object’s integration into the LiDAR scenes. Zhang et al. [17] utilized a conditional generative model that employs segmentation maps as a guiding tool to ensure the accurate generation of adverse effects, significantly improving the robustness of perception and object detection systems in autonomous vehicles under diverse and challenging conditions. Although robust LiDAR segmentation [18] employs domain-specific augmentation methods such selective jittering to address complicated spatial interactions in varied weather situations, it faces issues in preserving dataset quality and computational needs. Text3DAug represents a scalable LiDAR data augmentation method [19]. The prompting system generates annotated 3D instances from written descriptions and automates augmentation without intense labeling.

The most recent studies on LiDAR simulation techniques highlight a convergence of methodologies aimed at improving efficiency, realism, and applicability across various domains. Lopez et al. [20] focused on GPU-based LiDAR simulation to generate dense semantic point clouds for deep learning (DL), offering remarkable speed improvements and scalability in procedural environments. Building on such foundational simulations, Winiwarter et al. [21] introduced HELIOS++, a modular framework capable of simulating diverse LiDAR scenarios, such as terrestrial and airborne scanning, emphasizing the balance between computational efficiency and physical realism. While these studies emphasize physical modeling, Anand et al. [22] explored physics-informed deep learning by incorporating incidence angles to improve LiDAR intensity predictions using the U-NET and Pix2Pix architectures. Extending these advancements, Zyrianov et al. [23] presented LidarDM, a novel latent diffusion model that generates 4D layout-aware LiDAR sequences, revolutionizing virtual scene generation for autonomous driving. Complementing these innovations, Eggert et al. [24] leveraged game engines to create synthetic point clouds for industrial object detection, bridging the gap between real and simulated data. Together, these studies contribute towards versatile, scalable, and high-fidelity LiDAR simulation frameworks tailored to emerging applications in robotics, remote sensing, and AI.

Table 1 summarizes the current state-of-the-art LiDAR synthetic point cloud-generating methodology and its essential characteristics.

**Figure 1:**
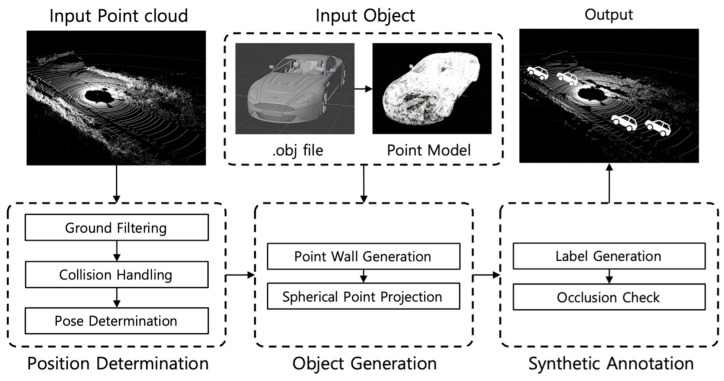
Overview of proposed framework.

Figure 1 presents an overview of the proposed framework. The framework proposed in this study operates in three parts: position determination, spherical point projection, and synthetic annotation modules. In the position determination module, the position and pose of the synthetic object to be generated are determined. The spherical point projection module generates synthetic object points using the spherical point projection and point-wall methods. In the synthetic annotation module, labels are attached to the projected points. The input object contains the point model, which uses an open-source point cloud library to convert each triangular polygon in the 3D shape of the freely distributed .obj file into a surface composed of points.

## 3. Proposed Method

### 3.1. Position Determination

#### 3.1.1. Ground Filtering

**Figure 2:**
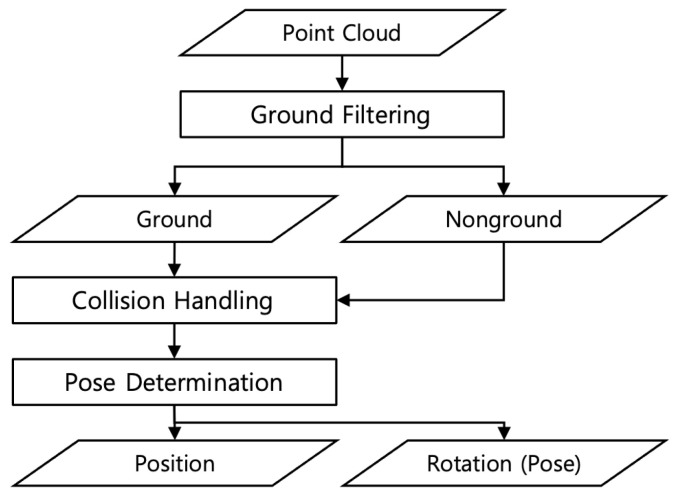
Main algorithm of position determination module.

The position determination module determines the position and orientation of the synthetic object to be generated. Ground filtering is used to separate the input data into ground and non-ground data, and random coordinates within the ground data are set as candidate positions for generation. Subsequently, collision handling is performed between these position candidates, and the orientation of the object at the final chosen position is determined. Figure 2 shows the main algorithm of the position determination module.

**Figure 3:**
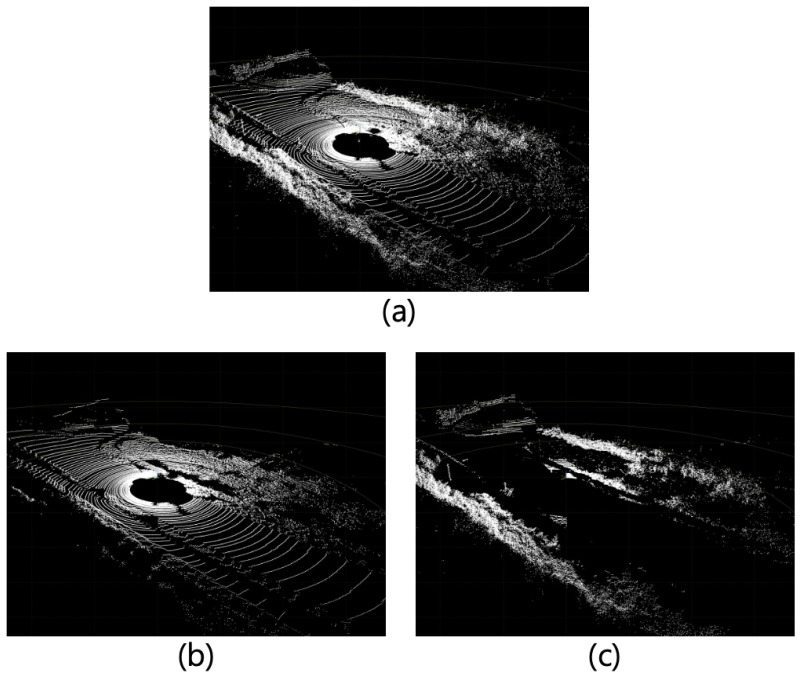
Ground filtering: (**a**) input LiDAR data; (**b**) filtered ground data of input point cloud; (**c**) filtered non-ground data.

This ground-filtering algorithm is used to determine an area in the input data where the synthetic object could be generated. This study uses the PatchWork++ [27] algorithm, which recognizes the ground by calculating the plane angle of a specific area. Subsequently, the ground data are randomly selected from the desired number of points and assigned as candidate positions for generation. Figure 3 shows the results of the ground-filtering algorithm.

#### 3.1.2. Collision Handling

**Figure 4:**
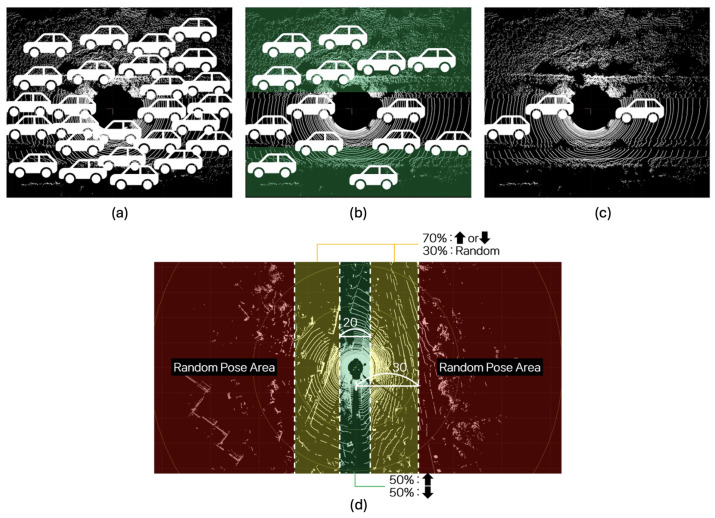
Collision handling and pose determination: (**a**) before collision handling; (**b**) after collision handling between virtual objects; (**c**) after collision handling between virtual objects and a non-ground point cloud; (**d**) vehicle pose distribution with respect to pose decision areas.

Collisions between synthetic objects (virtual objects) and real points (such as ground or non-ground points) are detected through a two-step process:

- Collision between virtual objects: After generating candidate coordinates for the synthetic objects (from the region of interest, or RoI, which is the ground point cloud), the algorithm first ensures that virtual objects do not overlap. It does so by selecting one candidate coordinate at random and removing all other coordinates within a certain distance, known as the “collision threshold”. This ensures that virtual objects are spaced out properly to prevent overlaps as shown in Figure 4b.
- Collision with non-ground points: Once the first collision detection (between virtual objects) is completed, the remaining candidate coordinates are checked for collisions with non-ground points (such as vegetation, sidewalks, etc.). This is carried out by comparing the coordinates with the non-ground point cloud, which was previously segmented using a ground segmentation algorithm. If any candidate coordinates are too close to non-ground points (within a specified distance), they are discarded. For the second step, the collision threshold used for virtual object-to-non-ground collision detection is half the value used for virtual object-to-virtual object collisions. This ensures a finer level of collision avoidance when checking proximity to non-ground features like vegetation or sidewalks as shown in Figure 4c. This approach of using distance-based thresholds for collision handling can be less precise when compared to synthetic mesh-based pruning. Although a mesh-based approach would improve placement precision, the computational trade-offs may not justify its use in large-scale dataset generation, particularly when speed and scalability are prioritized. Therefore, this limited approach comprising distance-based thresholds for collision handling is utilized, which will be replaced by mesh-based approaches in the future.

#### 3.1.3. Pose Determination

The pose determination algorithm determines the orientation of the synthetic object to be generated. This study uses the yaw value to determine the orientation; this parameter represents the rotation angle around the Z-axis in the 3D coordinate system and indicates the direction that the object is facing and the direction in which the vehicle is driving. The pose of the object is determined based on its position in the input data, considering the Korean road traffic infrastructure environment, and the following algorithm (Algorithm 1), which describes vehicles going straight, reversing, turning left, and turning right, as shown in Figure 4d. The pose determination module modifies the pose of the synthetic object by dividing the input point cloud space area by $O_{y}$, which is the lateral distance of the synthetic object *O* from the sensor (or the ego vehicle).

The yaw angle of synthetic objects (virtual objects) is determined through a multi-step process, taking into account the object’s location, its class (vehicle or non-vehicle), and the surrounding real-world vehicles. This function can be expressed by Equation (1):

(1) $$O_{Yaw}=\left\{\begin{array}{cc} 0\;\mathrm{or}\;\pi , & \mathrm{if}\;|O_{y}|\;<\;10\\ 0\;\mathrm{or}\;\pi \;\mathrm{or}\;Yaw_{rand}, & \mathrm{else}\;\mathrm{if}\;10\;<\;|O_{y}|\;<\;30\\ Yaw_{rand} & \mathrm{else}\; \end{array}\right.,$$

The yaw angle calculation is a two step process:

- Pose decision area: The initial yaw value is set based on the object’s Y-coordinate ($O_{y}$) in the LiDAR sensor’s coordinate system, divided into three areas:
  - Straight pose area ($O_{y}<10$): the yaw angle is set to 0 (same direction) or π (opposite direction), chosen randomly.
  - Intersection pose area ($10<O_{y}<30$): the yaw angle is set to 0, π, or a random value ($Yaw_{rand}$ between 0 and 2π).
  - Random pose area ($O_{y}\geq 30$): the yaw angle is set randomly between 0 and 2π.
- Update with nearby real vehicles: Once the yaw angle is assigned based on the pose decision area, it can be updated based on the orientation of nearby real vehicles. The input point clouds in this context are typically labeled with object categories such as “car”, “truck”, “pedestrian”, etc. If a real vehicle is within a certain proximity to the synthetic object, the yaw value of the virtual object is updated to match the yaw angle of the nearest real vehicle. Thereby, the yaw value is determined with reference to the position and orientation derived from the bounding box. This step reflects the real-world phenomenon where vehicles on the road often drive in the same direction (or opposite directions) in clustered groups, such as on highways or in dense traffic.

### 3.2. Object Generation

#### 3.2.1. Spherical Point Cloud Projection

The SPCP module is responsible for generating synthetic object points by projecting real-world data (LiDAR point clouds) into synthetic models. This process involves several key steps:

- Coordinate transformation: The module first converts the input LiDAR point cloud data from Cartesian (orthogonal) coordinates to spherical coordinates. This step is necessary for applying the spherical point-tracking technique.
- Spherical point tracking: Once the data are in spherical coordinates, the module uses spherical point tracking and point cloud wall creation techniques to generate a synthetic model of the virtual object. This process defines the structure of the synthetic object based on the real-world point cloud data.
- Projection and final transformation: After applying the spherical point-tracking method, the resulting synthetic model is converted back into Cartesian coordinates. This step finalizes the projection of the virtual object into the synthetic point cloud data, effectively augmenting the original data with the new object.
- Integration into synthetic annotation: The augmented point cloud data are then passed into the synthetic annotation module, which processes the data further to fit the required data format, reflecting object occlusion and other relevant information like object type, position, and orientation.

**Figure 5:**
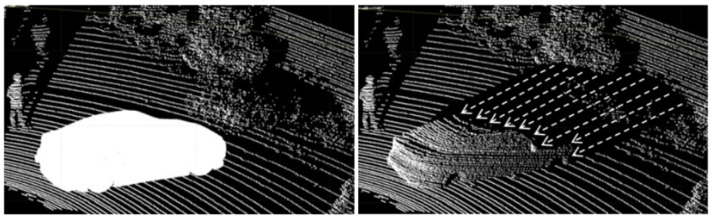
Spherical point projection method: white silhouette represents synthetic object onto which the real LiDAR points are projected (depicted as arrows).

Table 2 presents the data structures involved in generating synthetic object points by projecting real-world data into synthetic models. The proposed method uses the real acquired points to form synthetic object points. As a result, this work can more fully reflect the noise and loss distribution of real LiDAR sensors, and this is prominent for distant objects. Figure 5 shows the data before and after applying the proposed method.

**Figure 6:**
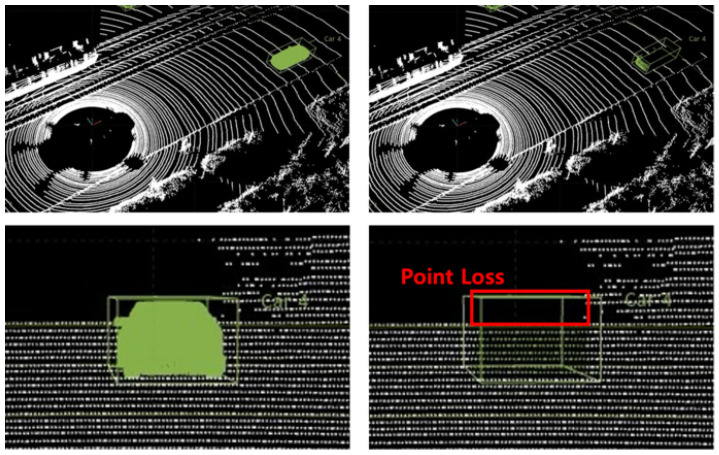
Point loss due to detection range of LiDAR sensor: green silhouette represents detected vehicle (car) when it is in the LiDAR’s detection range and point loss (red box) when car is out of the LiDAR’s detection range.

There are several types of losses in LiDAR point cloud data; however, the excessive loss mentioned here refers to the loss caused by rays radiating from the sensor that do not reflect off any object. In general, LiDAR sensors such as Velodyne HDL-32E manufactured by Velodyne Lidar, Inc., San Jose, CA, USA , with a horizontal resolution of $0.08^{\circ }∼1.33^{\circ }$ and a vertical resolution of $1.33^{\circ }$, have a detection range of approximately 100 m, a horizontal field of view of $360^{\circ }$, and a vertical field of view of $41.33^{\circ }$. If there are no objects within 100 m, the radiated rays do not return. This type of loss needs to be compensated for, as it would not have occurred if the synthetic object had originally been in that position. Figure 6 shows a case in which an excessive loss occurs in the shape of a synthetic object.

**Algorithm 1** Spherical Point Projection Algorithm

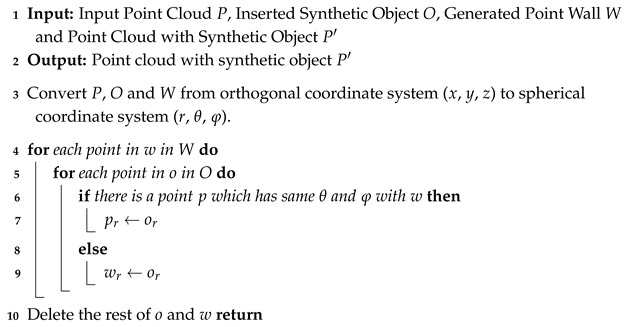



#### 3.2.2. Point Wall

**Figure 7:**
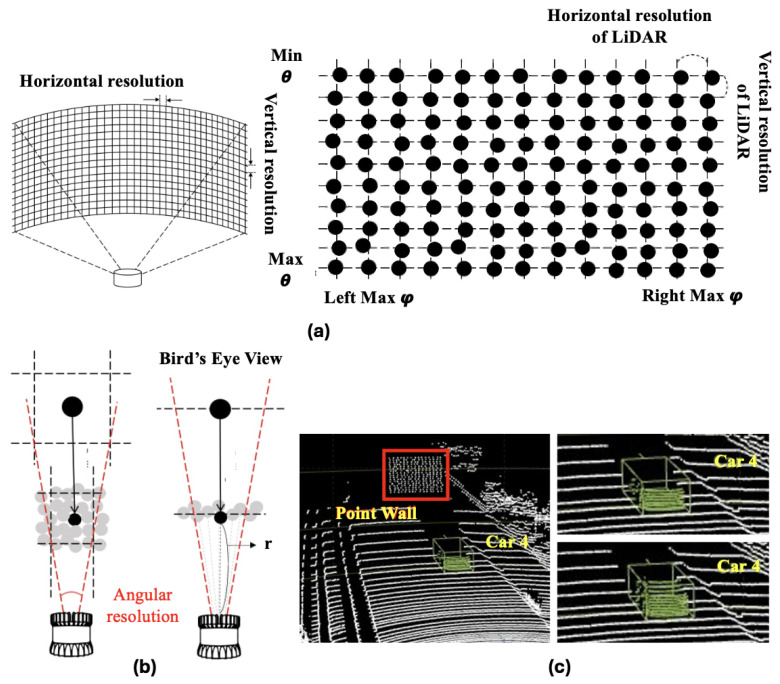
Point loss compensation by the point wall. (**a**) Appearance of the curved point wall generated by the proposed technique; (**b**) process of searching for point coordinates of a synthetic object point cloud model corresponding to a point in the input data and the arrows represent the perspective of normal view, bird’s eye view; (**c**) point loss compensation for the synthetic object point generation.

A point wall was considered to compensate for this type of loss by generating a point cloud wall with a resolution that matched the performance of the LiDAR sensor with the input data to fill in the lost parts. This allows for excessive losses that would not have occurred if the synthetic object had been in that position to be compensated for, and distant objects can be represented more realistically. The point walls reflect the horizontal, $res_{h}$, and vertical, $res_{v}$, resolution of the input point clouds. The indices, *i* and *j*, of the point wall are calculated within the $O_{\theta }$ and $O_{\varphi }$ ranges of the synthetic object data, as shown in Equation (2)

(2) $$\begin{array}{c} 0\leq i\leq \frac{max\left(O_{\theta }\right)-min\left(O_{\theta }\right)}{res_{h}}\\ 0\leq j\leq \frac{max\left(O_{\varphi }\right)-min\left(O_{\varphi }\right)}{res_{v}}, \end{array}$$

and to consider the scanning pattern of a LiDAR sensor, the resolutions and ranges in both the horizontal and vertical directions are parameterized along with the distance between the sensor and the point wall. Relevant parameters, such as $W_{r}=100$ (maximum distance range of a Velodyne HDL-32E LiDAR sensor) and horizontal and vertical spacings, are specified with respect to the spherical coordinate system values ($W_{\theta }$ and $W_{\varphi }$) of the point wall, as shown in Equation (3). The width of the curved point wall depends on the horizontal field of view (FoV) of the LiDAR sensor, as specified in Table 3 and shown in Figure 7b.

(3) $$\begin{array}{c} W_{\theta }=min\left(O_{\theta }\right)+\left(res_{h}\times i\right)+\epsilon_{h}\\ W_{\varphi }=min\left(O_{\varphi }\right)+\left(res_{v}\times j\right)+\epsilon_{v}\\ W_{r}=100 \end{array}$$

are calculated using this index. Next, the horizontal noise and vertical noise, $\epsilon_{h}$ and $\epsilon_{v}$, are added, similarly to the noise of the real sensor. Figure 7 shows the recovery of the loss of the synthetic object using a point wall. Inaccurate placement of the point wall can be avoided by using the input parameters of the sensor. The noise level in the position of the point cloud data is within 30% of the horizontal and vertical resolutions, which mitigates the possibility of generating duplicate point clouds in the same location. However, this study does not consider return losses according to the reflected intensity, which demands further investigation regarding materials and reflectivity.

### 3.3. Synthetic Annotation

**Figure 8:**
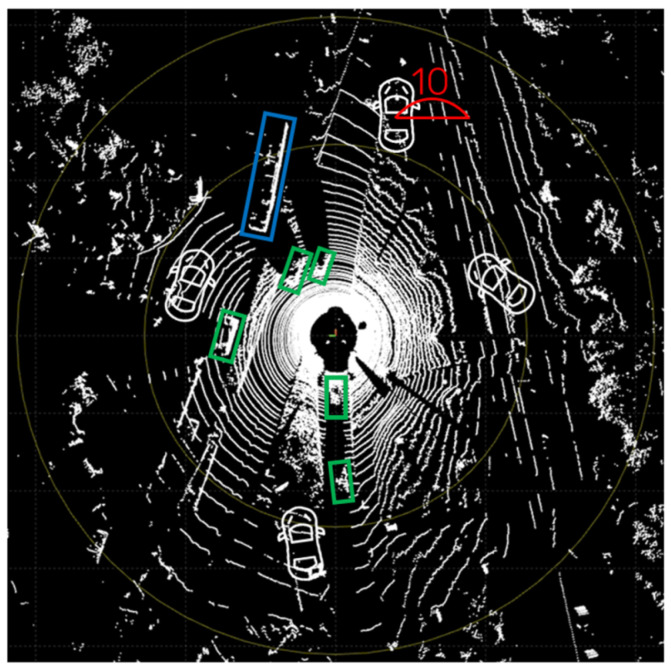
Adapting object rotation and position based on proximity and default parameters: green represents car objects, blue represents bus objects and red area indicates a horizontal range of ±10 m within which the synthetic cars (white cars) are generated.

The synthetic annotation module generates labeling information for the generated synthetic object points. Labeling data for 3D objects typically include information such as the object’s position, size, pose, and occlusion and are automatically generated using information about the generated synthetic object, as shown in Figure 8.

Additional occlusion handling is performed because existing real objects can be affected by synthetic object points. For example, if a synthetic car is generated in front of a real car, it is occluded, which must be reflected in the labeling data. Through this process, the labeled data for synthetic object points were recorded and saved as a text file.

The labeling data consist of the same format as the KITTI 360 dataset, which is the most commonly used 3D object detection dataset. Since the KITTI 360 dataset includes data in which 2D data and 3D data are fused, it is characterized by storing 3D location information data as 2D data. It basically includes the location, size, and direction data of a 3D cuboid and stores data on how much the object is hidden from other objects and how much it is cut off by the sensor’s field of view. Table 4 shows the format of the KITTI 360 dataset’s labeling data.

The ‘truncated’ and ‘occluded’ aspects of the synthetic object are calculated through occlusion handling, and information about the synthetic object generated in the data is obtained before the synthetic object is created, which is then modified when the real object is affected by the synthetic object to reflect this. The completed labeling data are written and stored as a .txt format file, just like the labeling file in the KITTI 360 dataset.

## 4. Experimental Results

### 4.1. Experimental Environment

#### Dataset and Model

For the synthetic LiDAR point cloud generation experiment, the KITTI 360 [28] and nuScenes [29] datasets were used. The KITTI 360 dataset is one of the most commonly used autonomous driving datasets and includes 3D LiDAR data. The LiDAR sensor used for data acquisition was the HDL-64E model developed by Velodyne, which is a 64-channel model. The nuScenes dataset is an autonomous driving dataset that includes LiDAR data and uses the HDL-32E model from Velodyne, a 32-channel LiDAR. These two datasets, shown in Table 3, were chosen for the experiment because they were acquired using LiDAR sensors with channels different from those of Velodyne. The datasets include object bounding boxes and AP3D (%) evaluation metrics, assessing object detection performance across easy, moderate, and hard scenarios, considering occlusion and object size.

For the deep learning model training experiment, PointPillars [30], PV-RCNN [31], and Voxel R-CNN [32] models were used as 3D object detection models. The PointPillars model is a network that shows outstanding computational speed owing to its pillar-shaped feature extraction and is still widely used due to its real-time performance. Both the PV-RCNN and Voxel R-CNN models achieved state-of-the-art (SOTA) results, demonstrating superior performance compared to previous research.

### 4.2. Synthetic LiDAR Point Cloud Generation

**Figure 9:**
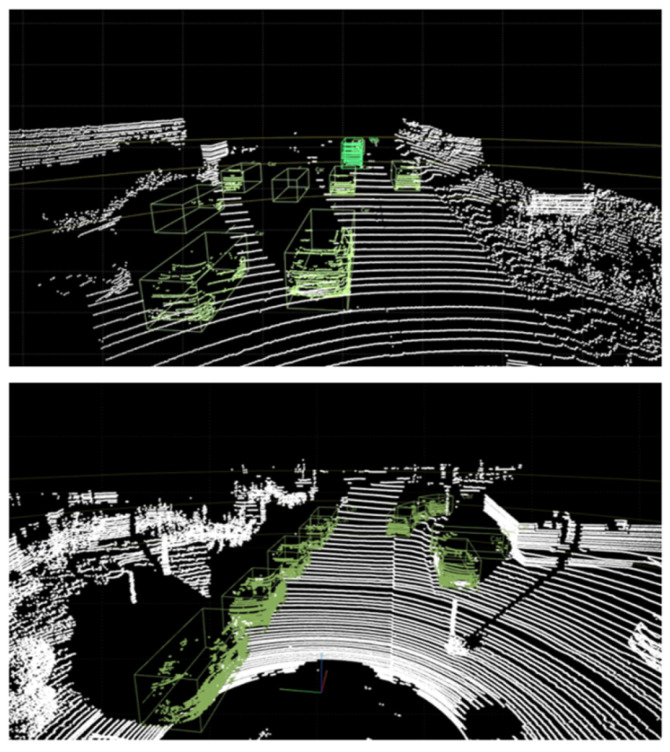
Platooning situation that appears in the KITTI 360 dataset: green represents car objects, and light green represents van objects.

**Figure 10:**
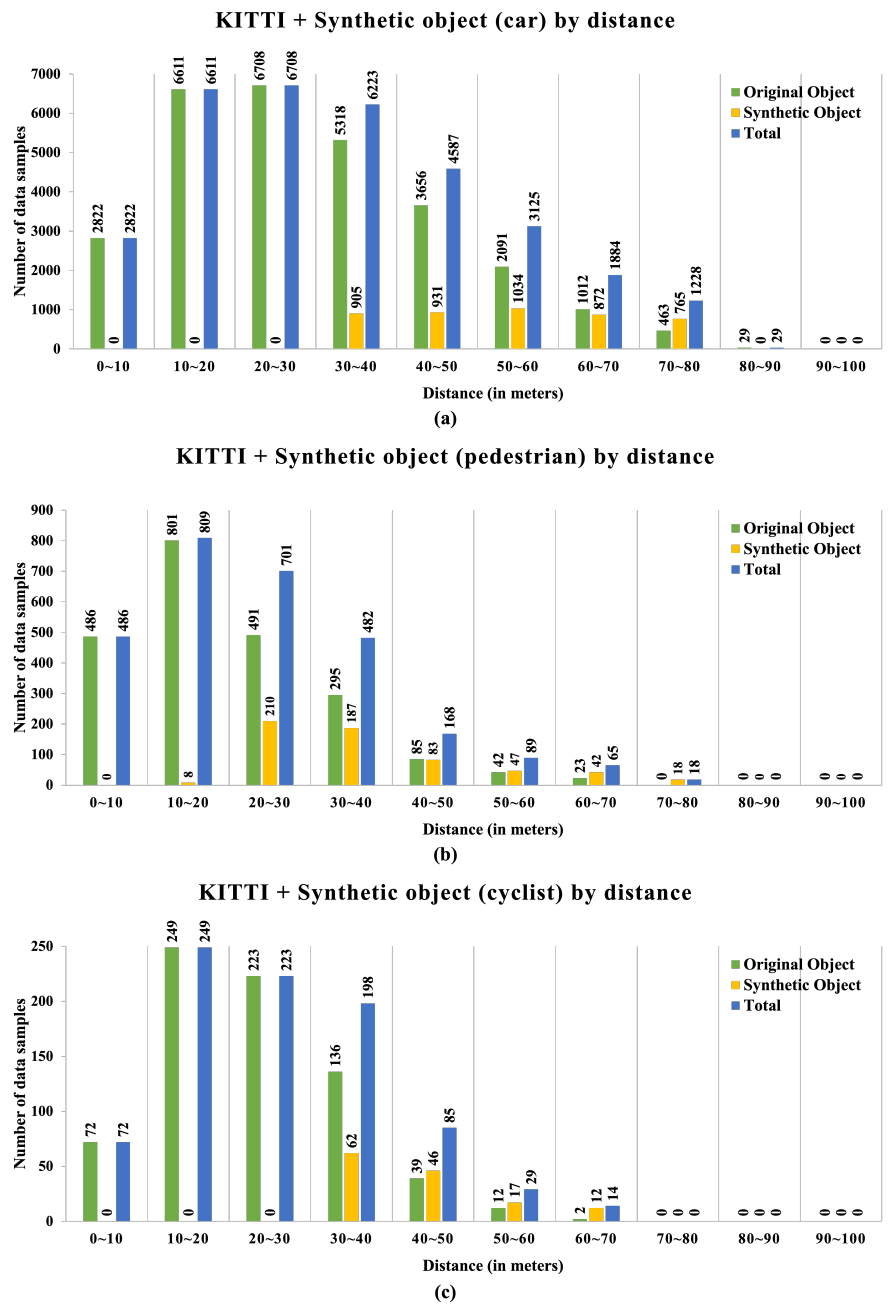
The proportion of data generated by each category—(**a**) car, (**b**) pedestrian, and (**c**) cyclist—at different distances from the original data.

The experiment was divided into a car class generation experiment, which accounted for the majority of the experiment, and pedestrian and cyclist class generation experiments. In addition, a generation experiment was conducted to verify the representation of platooning, as shown in Figure 9, using the synthetic object position determination module of the proposed framework and the data distribution with object distance, as shown in Figure 10.

#### 4.2.1. Car Class

**Figure 11:**
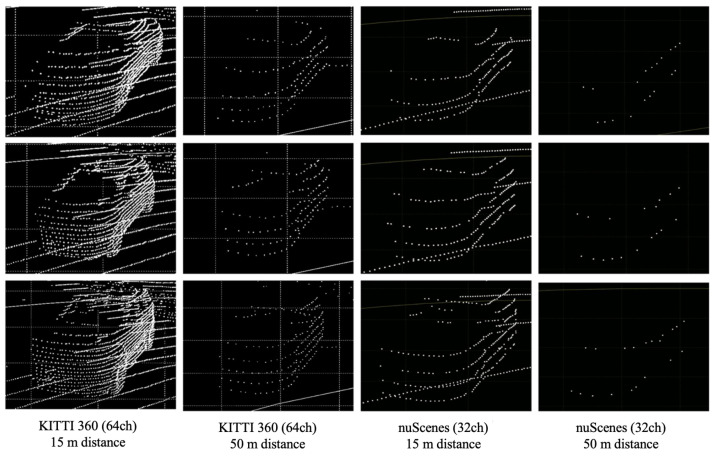
Results of synthetic LiDAR point cloud generation experiment for car class.

The car class represents the most frequently observed objects in autonomous driving datasets. The experiment was divided into near distances of over 15 m and far distances of over 50 m, generating synthetic object points on both the 64-channel LiDAR data from the KITTI 360 dataset and the 32-channel LiDAR data from the nuScenes dataset. Figure 11 shows the resulting image of the synthetic LiDAR point cloud generation for the car class.

**Figure 12:**
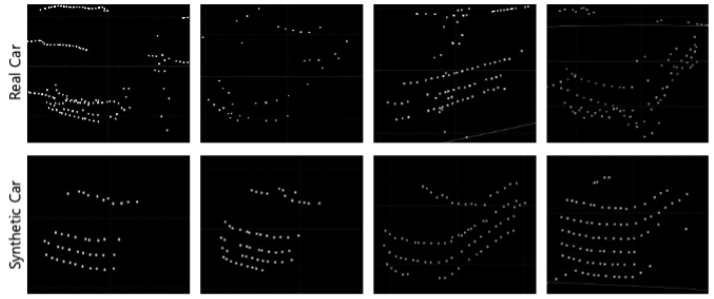
Comparison of distant synthetic car objects generated using the proposed method with real distant car objects.

**Figure 13:**
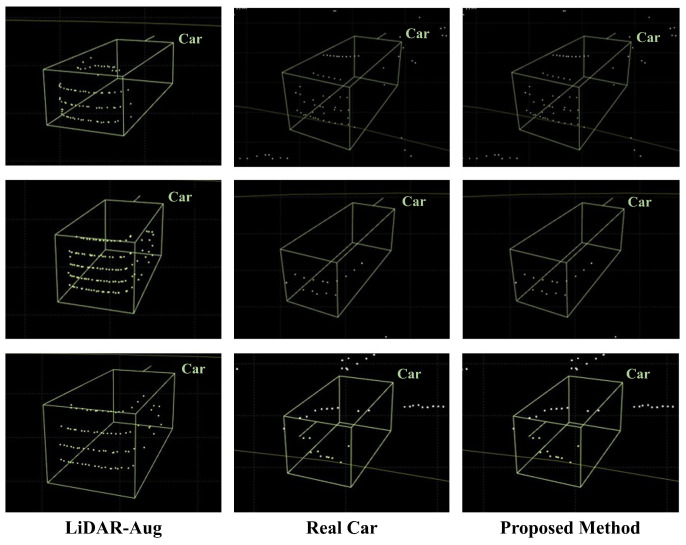
Comparison of realism factor between LiDAR-Aug and the proposed method.

The results of creating synthetic car class objects using the proposed framework showed that it is capable of generating synthetic object points with shapes similar to those of real objects for both near and far distances. The shape of objects in LiDAR point cloud data varies depending on the channel and the performance of the sensor. This experiment proved that these variations could be significantly well represented by the shape of the objects. Figure 12 compares the shapes of the generated synthetic car objects with those of the real objects at the same distance. Synthetic LiDAR point cloud generation occurred at distances greater than 50 m and showed the appearance of real vehicles at similar distances as the synthetic vehicles generated by LiDAR-Aug and the proposed method. The LiDAR-Aug method was implemented based on the proposed method, and synthetic object points reflecting Gaussian noise were generated. Figure 13 compares the shapes of the synthetic object points generated by the existing LiDAR-Aug and proposed frameworks. The experimental results confirmed that our method represents long-distance objects that are more similar to real ones.

#### 4.2.2. Pedestrian and Cyclist Classes

**Figure 14:**
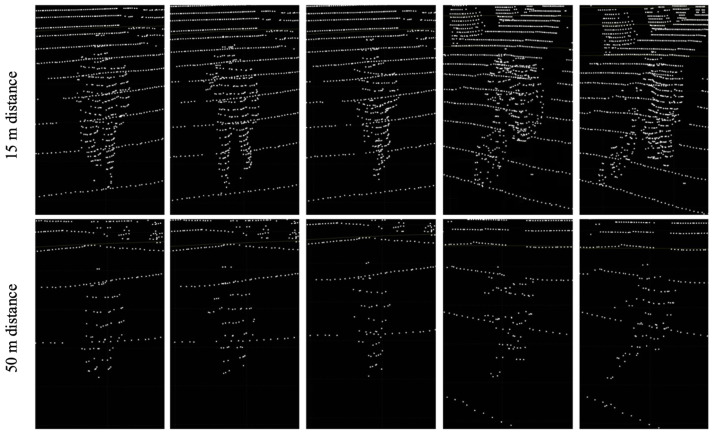
Experimental results of synthetic LiDAR point cloud generation (pedestrian and cyclist classes).

This study also conducted generation experiments for the pedestrian and cyclist classes, which are the most used classes after the car class in autonomous driving datasets. Similarly to the car class, synthetic object points were generated at near distances of 15 m and far distances of 50 m, and it was confirmed that the synthetic object points generated at each distance showed shapes similar to those of real objects. Although the shapes of the objects were not as distinct as those of the larger car class because of their smaller size, this proved the method’s ability to represent the shape of the objects differently depending on the distance. Figure 14 shows the resulting images of the synthetic LiDAR point cloud generation for the pedestrian and cyclist classes.

#### 4.2.3. Platooning

**Figure 15:**
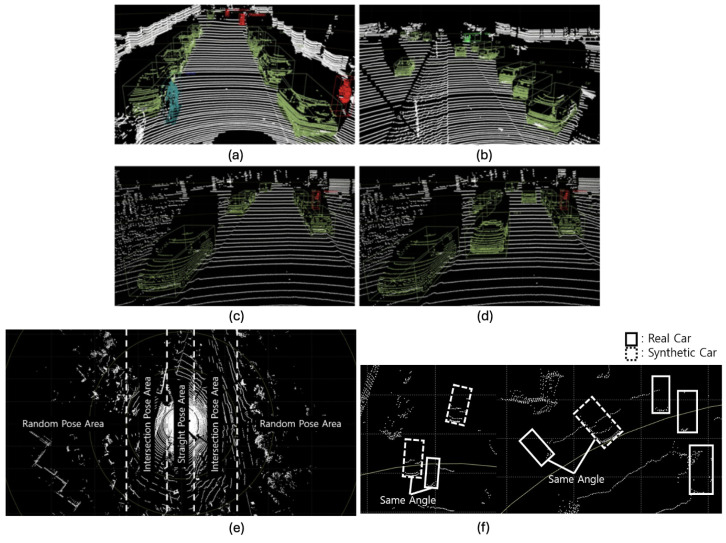
Platooning represented by proposed method where green represents car objects, light green represents van objects, blue represents cyclist objects, and red represents pedestrian objects. (**a**,**b**) Frame presenting the platooning situation included in the KITTI 360 dataset; (**c**) original input LiDAR scene; (**d**) output scene with 2 synthetic car objects; (**e**) pose decision areas for platooning with respect to a real vehicle; (**f**) pose of a synthetic object determined by a nearby real vehicle for platooning.

Figure 9 shows the platooning situation that appears in the real KITTI 360 dataset, and the other image shows a situation in which the synthetic object points generated through the proposed framework depict platooning. In the figure, two synthetic object points are generated, both of which follow the direction of nearby real objects by following two steps: (1) calculating the pose decision area based yaw value determination and (2) updating the pose of the synthetic object to point toward a nearby real vehicle’s pose, as stated in Section 3.1.3. In summary, the yaw angle is assigned based on the pose decision area, and then it can be updated based on the orientation of nearby real vehicles. If a real vehicle is within a certain proximity to the synthetic object, the yaw value of the virtual object is updated to match the yaw angle of the nearest real vehicle, allowing for a platooning process, as shown in Figure 15f. However, the current study should further explore complex platooning mechanisms besides the angle alone to implement better pose estimation techniques. This aspect is considered as a potential future scope in terms of platooning scenarios.

### 4.3. Deep Learning Model Training

The KITTI 360 dataset is a large-scale autonomous driving dataset consisting of approximately 15,000 frames of data in the form of a fusion of 2D image data and 3D LiDAR sensor data. Approximately 15,000 frames of data are provided in the form of Train, Val, and Test sets in a ratio of 2:1:1. Most models in the 3D object detection field use the KITTI 360 dataset to demonstrate object recognition performance, and a benchmark suite is provided for this purpose so that developers of object recognition deep learning models can access and utilize it. To confirm that the synthetic object points generated through the proposed framework can be effectively used for deep learning model training on RAM 8 GB Intel i5/GTX 1080 8 GB, datasets augmented with synthetic object points were used to train various deep learning models and evaluate their performance. The deep learning model training experiments were conducted separately using the datasets augmented with the car class and those augmented with the pedestrian and cyclist classes.

#### 4.3.1. Car Class

A dataset augmentation experiment using synthetic object points from the car class was conducted by training various 3D object detection models on the augmented dataset and measuring the improvement in training performance. The KITTI 360 dataset was used, and the PointPillars, PV-RCNN, and Voxel R-CNN models were used as the deep learning models. The performance of the proposed framework was evaluated in comparison with an existing research method, LiDAR-Aug. Table 5 lists the car class performances of the proposed framework. Since Voxel-RCNN was published before LiDAR-Aug, this item is vacant, and the training performance of the dataset augmented with the proposed framework improved compared with the previous ones for all three selected models. The extent of improvement in training performance showed a trend in which it was higher when the original performance was lower and somewhat lower when the original performance was higher. It was also observed that the performance of the proposed framework improved compared to that of LiDAR-Aug for all the models. The performance of LiDAR-Aug for the voxel R-CNN model was not included as it was not presented in this paper.

#### 4.3.2. Pedestrian and Cyclist Classes

The dataset augmentation experiment for the pedestrian and cyclist classes was conducted in a similar manner using the augmented KITTI 360 dataset to train the PointPillar and PV-RCNN models and to calculate the improvement in training performance. The performance of the dataset augmentation carried out via the proposed framework was also validated by comparing it with the performance of LiDAR-Aug. Table 6 and Table 7 list the performance of the proposed framework for the pedestrian and cyclist classes. The performance of the proposed framework for the pedestrian and cyclist classes was slightly improved compared with the original dataset. However, when compared to the LiDAR-Aug method, the proposed framework did not outperform LiDAR-Aug in every case. The performance of the cyclist class was not included as it was not presented in the LiDAR-Aug paper.

Interestingly, although the dataset was augmented for the pedestrian and cyclist classes, the training performance for the car class improved. This is interpreted as dataset augmentation mitigating the object imbalance between classes that existed because the original dataset had fewer pedestrian and cyclist class objects than car class objects, thereby enhancing the learning performance for the car class as well.

## 5. Limitations and Future Work

The proposed framework consists of several limitations, such as the fact that the performed experiments primarily focused on evaluating metrics such as accuracy (mAP) in favor of the training process. Dataset diversification must be carried out by introducing vast datasets for LiDAR and employing the proposed framework to improve training performance. However, including a variety of metrics such as the real-to-synthetic noise distribution and testing for realism would favor the overall integration of the framework into the training process. Additionally, other evaluation models could be used aside from those included in the current study, which were limited to PointPillar, PV R-CNN, and Voxel R-CNN. Due to the lack of open-source SOTA resources for reproducibility, this study employed the KITTI dataset to perform the evaluation, alongside LiDAR-Aug. Subsequent research may extend this process to produce integrated 2D and 3D virtual entities with calibrated LiDAR and picture data, thereby reducing the time and resources necessary for synthetic LiDAR point cloud generation. Also, this study did not consider return losses according to the reflected intensity, which requires further investigation regarding materials and reflectivity. The idea of reflectance was not explored in this study, which is a shortcoming, and it should be explored in future work. Additionally, various complex platooning mechanisms must be explored with better pose estimation variables besides the angle.

## 6. Conclusions

The proposed framework includes a module to determine the pose of synthetic object points, along with an automated system capable of representing both near and distant synthetic object points, as well as platooning scenarios for vehicles on the road. The proposed framework was assessed by qualitative and quantitative performance analyses on the synthesis of objects within an established dataset, namely KITTI. The integration of the synthetic object through this framework in the augmented dataset demonstrated that synthetic object points can be efficiently utilized in training deep learning models for 3D object detection applications. This study showed that the proposed framework can accurately represent distant objects and produce synthetic object points that closely align with real-world distributions, in contrast to the existing LiDAR-Aug technique. The performance of the proposed framework was evaluated on various 3D detection models, such as PointPillars, PV-RCNN, and Voxel R-CNN, for the KITTI dataset. The results indicated an increase in mAP (mean average precision) by 1.97%, 1.3%, and 0.46% from the original dataset values of 82.23%, 86.72%, and 87.05%, respectively. The proposed method has to deal with return loss in new projected points, which is a shortcoming at this stage. Future investigations may expand this methodology to generate integrated 2D and 3D virtual entities with calibrated LiDAR and image data, thereby minimizing the time and resources required for AI dataset generation and enhancing autonomous driving technology.

## Figures and Tables

**Table 1:** Insights into object generation and data augmentation for 3D object detection networks for LiDAR data.

Research Study	Methodology	Key Aspects	Limitations
Esmoris et al. [1]	Trains models with virtual laser scanning data	Automated scene and model training	Limited to specific real data applications
Yin et al. [7]	Extended DART model with Monte Carlo methods for satellite LiDAR simulation	Efficient scattering models; supports data fusion with other sensors	Focuses on vegetation and urban scenes
Zhao et al. [16]	LiDARsim uses real data, ray casting, and neural networks	Realistic LiDAR for autonomous testing	High fidelity needed, weather simulation issues
Zhang et al. [17]	Conditional generative model with segmentation maps	Large dataset, domain adaptation strategies.	Cannot generalize to more severe settings
Park et al. [18]	Generative models for long-tail object recognition	Generative augmentation for minority classes.	Focuses only on object recognition
Reichardt et al. [19]	Virtual laser scanning for semantic segmentation	Automated training, real-world data reliance.	Accuracy gaps, dynamic scene challenges
Lopez et al. [20]	GPU-based LiDAR simulator generates dense semantic point clouds for DL training	High speed (99% faster); large-scale labeling; procedural scene generation	Limited to procedural or static environments
Winiwarter et al. [21]	HELIOS++ simulates terrestrial, airborne, and mobile LiDAR with modular scene modeling	Handles vegetation, supports Python, has fast runtime, and creates training data	Slightly less accurate than ray-tracing models
Anand et al. [22]	Physics-informed DL for LiDAR intensity simulation using U-NET and Pix2Pix architectures	Adds incidence angle as input; improves intensity prediction accuracy	Lacks material property integration
Zyrianov et al. [23]	LidarDM generates realistic, layout-aware, temporally coherent 4D LiDAR sequences	4D generation; driving scenario guidance; high realism for simulations	Not real time; no intensity modeling yet
Eggert et al. [24]	Synthetic point cloud generation using Unreal Engine for object detection	High-quality clouds; suitable for industrial datasets	Sparse datasets; lacks specific real-world equivalency
Manivasagam et al. [25]	Simulates LiDAR with real data, simulations, and ML	Realistic LiDAR simulations	Requires large datasets, domain challenges
Fang et al. [13]	Rendering-based LiDAR augmentation	Point distribution representation	Low performance for long-distance objects
Xiao et al. [14]	SynLiDAR dataset creation, translation via PCT	Large synthetic dataset, transfer learning	Focused on segmentation, overfitting risk
Xiang et al. [15]	Generative models for LiDAR object recognition	Synthetic point clouds, minority class focus	Limited generalization, needs tailored models
Li et al. [26]	PointAugment auto-optimizes point cloud samples via adversarial learning	Sample-specific augmentation; improves shape classification	Limited to certain transformations

**Table 2:** Data structures involved in projecting real-world data into synthetic models.

Values	Category	Data	Type
1	Class	Describes the type of object	string
3	Position	3D object location in LiDAR coordinates (in meters) Ex. [$Pos_{x}$, $Pos_{y}$, $Pos_{z}$]	float[]
3	Rotation	3D object rotation in LiDAR coordinates Ex. [0, 0, Yaw]	float[]
3	Dimension	3D object dimensions in LiDAR coordinates (in meters) Ex. [$Dim_{x}$, $Dim_{y}$, $Dim_{z}$]	float[]
1	Occluded	Integer (0,1,2,3) indicating occlusion state: 0 = fully visible 1 = partly occluded 2 = largely occluded 3 = unknown	int

**Table 3:** Performance of Velodyne HDL-64E and HDL-32E LiDAR sensors manufactured by Velodyne Lidar, Inc., San Jose, CA, USA.

		Velodyne HDL-64E (KITTI 360 Dataset)	Velodyne HDL-32E (nuScenes Dataset)
Range		∼120 m	∼100 m
Resolution	Horizontal	0.08°	0.08∼1.33°
	Vertical	0.4°	1.33°
Field of View	Horizontal	360°	360°
	Vertical	26.8°(−24.8∼+2)	41.33°(−30.67∼+10.67)

**Table 4:** Three-dimensional object detection labeling data format for Kitti 360 dataset.

Values	Name	Description
1	Type	Describes the type of object: ‘Car’, ‘Van’, ‘Truck’, ‘Pedestrian’, ‘Person_sitting’, ‘Cyclist’, ‘Tram’, ‘Misc’ or ‘Dont Care’
1	Truncated	Float from 0 (non-truncated) to 1 (truncated), where truncated refers to the object leaving the image boundaries
1	Occluded	Integer (0,1,2,3) indicating occlusion state: 0 = fully visible 1 = partly occluded 2 = largely occluded 3 = unknown
1	Alpha	Observation angle of object, ranging [−pi, pi]
4	Bbox	2D bounding box of object in the image (0-based index) : contains left, top, right, and bottom pixels
3	Dimensions	3D object dimensions: height, width, and length (in meters)
3	Location	3D object location x, y, z in camera coordinates (in meters)
1	Rotation_y	Rotation, ry, around Y-axis in camera coordinates [−pi, pi]

**Table 5:** Evaluation results of proposed framework for the car class on the KITTI validation dataset.

Methods	AP3D (%)			mAP
	Easy	Moderate	Hard	
PointPillars with KITTI	85.41	73.59	68.76	75.92
PointPillars with LiDAR-Aug	87.75	77.83	74.90	80.16
PointPillars with proposed work (Ours)	88.75	80.43	77.52	82.23
PV-RCNN with KITTI	88.86	78.83	78.30	82.00
PV-RCNN with LiDAR-Aug	90.18	84.23	78.95	84.45
PV-RCNN with proposed work (Ours)	92.64	84.54	82.98	86.72
Voxel R-CNN with KITTI	92.24	85.01	82.51	86.59
Voxel R-CNN with LiDAR-Aug	NB	NB	NB	NB
Voxel R-CNN with Proposed (Ours)	92.67	85.34	83.13	87.05

**Table 6:** Evaluation results of proposed work for the pedestrian class on KITTI validation dataset using pointpillars.

Methods	AP3D (%)								
	Car			Pedestrian			Cyclist		
	Easy	Moderate	Hard	Easy	Moderate	Hard	Easy	Moderate	Hard
PointPillars with KITTI	85.41	73.59	68.76	47.51	43.82	42.20	84.64	64.26	60.69
PointPillars with LiDAR-Aug	87.75	77.83	74.90	59.99	55.15	52.66	-	-	-
PointPillars with Proposed work	87.99	78.42	75.35	56.28	50.5	46.07	84.68	64.63	61.12

**Table 7:** Evaluation results of proposed framework for the pedestrian class on KITTI validation dataset using PV-RCNN.

Methods	AP3D (%)								
	Car			Pedestrian			Cyclist		
	Easy	Moderate	Hard	Easy	Moderate	Hard	Easy	Moderate	Hard
PV-RCNN with KITTI	88.86	78.83	78.30	60.56	53.75	51.90	90.24	71.83	68.33
PV-RCNN with LiDAR-Aug	90.18	84.23	78.95	65.05	58.90	55.52	-	-	-
PV-RCNN with Proposed (Ours)	92.00	84.68	82.67	66.41	59.45	53.62	90.85	72.42	69.04

## Data Availability

The data underlying the conclusions of this article will be made available by the corresponding author upon reasonable request.
